# Policy options on a sustainable path for clinical trials 

**DOI:** 10.2471/BLT.26.295840

**Published:** 2026-03-01

**Authors:** Gabrielle Samuel

**Affiliations:** a Global Health and Social Medicine, King’s College London, Bush House North East Wing, 30 Aldwych, WC2B 4BG, London, England.

Medical research is a cornerstone of global health progress and a vital mechanism for developing new therapies and preventing and managing health burdens. However, as this theme issue of the *Bulletin of the World Health Organization* demonstrates, this research is associated with environmental harms.^1^^–^^4^ Authors have emphasized the research community’s collective responsibility to ensure its practices do not exacerbate environmental harms, yet large-scale culture change in research practice is undermined by a lack of coherent, mandatory policy.

This policy gap is pronounced for large-scale clinical trials, whose effects are exacerbated by additional environmental harms associated with international air travel of trial actors; the manufacture and shipment of investigational products, materials and equipment; and the shipment of clinical trial samples to centralized sites for analysis.^5^^–^^8^ In addition, climate-related disruptions such as flooding, storms and wildfires may increasingly pose risks to clinical trial site operations, disrupt supply chains and force site closures, compromising data collection and jeopardizing trial participant safety.

While increasing interest in greening clinical trials has led to the development of frameworks and methods to help trialists quantify and potentially minimize environmental harms,^8^ these initiatives lack clear policy support and standardized guidance from policy-makers, regulators and funders. Here, I suggest five key areas for policy implementation, which are likely to also be relevant to the wider medical research community.

First, regulatory bodies and funders should explicitly link environmental co-benefits to existing clinical trial research quality and efficiency standards, such as those within the International Council for Harmonization *Guideline for good clinical practice*.^9^ Methodologically poor research leads to a waste of resources, and therefore environmental harm. National regulatory authorities should also ensure that clinical trial protocols and findings are registered on international registries to guarantee that trialists have access to up-to-date evidence when designing trials, thereby avoiding research waste. Funders should contribute by ensuring that their research approval committees comprise sufficient expertise to judge whether trial proposals have assessed and demonstrated a sufficient gap in the evidence.

Second, funding bodies and regulatory agencies should require environmental considerations as a compulsory component of clinical trial submissions to ensure clinical trial sustainability-by-design. This requirement is similar to gender or equity considerations, which are now integral to protocol development, and should be harmonized globally through the *Guideline for good clinical practice*.^9^ Regulatory authorities should also publish aligned regulation, if not already developed, around sustainable procurement, as well as guidance around where and when it is appropriate to reuse, recycle and use shared equipment and reagents during trials. Funders can query budgets that comprise large and/or digital equipment, as its manufacture is a considerable source of environmental harm.

Third, regulators and funders must provide clear guidance on how trialists can reduce environmental harms in a way that does not jeopardize regulatory compliance. Trialists and sponsors operate in a risk-averse environment: they will not confidently adopt methods that might reduce environmental harms if they fear these approaches could create regulatory ambiguity or delay approvals. These methods include decentralized trial designs, remote monitoring or local manufacturing of materials and reagents.^5^ Regulatory bodies can provide the certainty that environmentally sound practices are not just permitted but encouraged, where research integrity and participant safety are not compromised. Moreover, some decentralized practices also improve participant inclusion through increased access to participation.

Fourth, in the context of climate instability, regulators and funding bodies can require trialists, particularly those conducting large studies in climate-vulnerable regions, to incorporate a mandatory climate risk assessment. This assessment should include contingency protocols for participant management and data-collection procedures in the event of a climate and/or environment emergency, so that the trial is not compromised and participants are protected, thereby fulfilling a core element of their ethical duty of care.^5^

Finally, achieving lasting transformation requires strategic investment in strengthening local and regional research ecosystems so that reliance on long-distance supply chains and sample flows can be reduced.^10^ Relevant ministries can work towards improving supply chain resilience within national infrastructures and promote local capabilities to collect and analyse clinical trial findings, as well as promote capacity development for training and certification of trial monitors in local contexts. Developing local capacity and training local trial personnel responsible for monitoring is key for supporting virtual visits and meetings, thereby reducing environmental harms linked to air travel.

By implementing these five areas of focused and actionable policies, national authorities, regulatory bodies and funders can steer the sector towards a sustainable path for clinical trials.
